# Margination of micro- and nano-particles in blood flow and its effect on drug delivery

**DOI:** 10.1038/srep04871

**Published:** 2014-05-02

**Authors:** Kathrin Müller, Dmitry A. Fedosov, Gerhard Gompper

**Affiliations:** 1 Theoretical Soft Matter and Biophysics, Institute of Complex Systems and Institute for Advanced Simulation, Forschungszentrum Jülich, 52425 Jülich, Germany

## Abstract

Drug delivery by micro- and nano-carriers enables controlled transport of pharmaceuticals to targeted sites. Even though carrier fabrication has made much progress recently, the delivery including controlled particle distribution and adhesion within the body remains a great challenge. The adhesion of carriers is strongly affected by their margination properties (migration toward walls) in the microvasculature. To investigate margination characteristics of carriers of different shapes and sizes and to elucidate the relevant physical mechanisms, we employ mesoscopic hydrodynamic simulations of blood flow. Particle margination is studied for a wide range of hematocrit values, vessel sizes, and flow rates, using two- and three-dimensional models. The simulations show that the margination properties of particles improve with increasing carrier size. Spherical particles yield slightly better margination than ellipsoidal carriers; however, ellipsoidal particles exhibit a slower rotational dynamics near a wall favoring their adhesion. In conclusion, micron-sized ellipsoidal particles are favorable for drug delivery in comparison with sub-micron spherical particles.

The use of targeted micro- and nano-carriers for the delivery of imaging agents and drugs provides a promising strategy for early detection and treatment of diseases, e.g., of cancer^1^,^2^. However, the design of particles carrying different contrast agents and drugs as well as their physical delivery are very challenging tasks. Micro- and nano-particle fabrication, which needs to address several issues such as bio-compatibility, durability, binding to specific targets, and the ability of controlled release, has been strongly advanced in recent years^3^,^4^,^5^,^6^,^7^. Nevertheless, the development of efficient strategies for the delivery of carriers, including their distribution in the organism following systemic administration^8^ and their transport through biological barriers^8^,^9^,^10^ (e.g., microvascular walls, interstitial space, and cell membranes), requires a much more detailed understanding of the relevant physical and biological mechanisms^2^,^8^,^11^,^12^.

Successful delivery of micro- and nano- carriers strongly depends on their efficient binding to specific targeted sites. Consequently, the distribution of carriers within vessel cross-sections plays an important role, since binding of carriers is only possible in case of direct particle-wall interactions. The cross-sectional distribution of micro- and nano-particles depends on several relevant parameters, which concern blood flow properties (such as flow rate, red blood cell deformability, and hematocrit – the volume fraction of red blood cells), vessel size, and particle characteristics (such as size, shape, and deformability). The migration of various suspended particles or cells toward walls in blood flow, which is often referred to as *margination*, has been observed experimentally for white blood cells^13^,^14^, platelets^15^,^16^, and rigid micro-particles^17^,^18^. Particle margination is mediated by red blood cells (RBCs), which migrate to the vessel center^19^ due to hydrodynamic interactions with the walls (called lift force)^20^,^21^ leading to a RBC-free layer near the walls. More precisely, the occurrence of margination is a consequence of the competition between lift forces on RBCs and suspended particles, and their interactions in flow^22^. However, the dependence of margination efficiency on particle size and shape remains largely unexplored so far.

The role of particle size and shape in the efficient delivery is a multi-faceted problem. Large enough particles with a characteristic diameter (*D_p_*) greater than about 4 μm may become trapped in the smallest capillaries of the body^23^. In addition, recent experiments suggest that large particles with 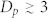 are subject to an enhanced phagocytosis^24^. However, recent microfluidic experiments^25^ have shown that spheres with the size of 2 μm show a significantly higher adhesion density than particles with a size of 200 nm and 500 nm. Other experiments^26^ indicate that liposomes with *D_p_* < 70 nm and *D_p_* > 300 nm have shorter circulation times than those having an intermediate size of *D_p_* ≈ 150–200 nm. Furthermore, nano-particles with a size below 20–30 nm are rapidly excreted through the kidneys^27^. Experiments with discoidal particles^28^ have shown that they accumulate in the organs better than their spherical counterparts; however, particle internalization by macrophages appears to be worse for elongated particles^29^. Adhesion of different particles has been studied experimentally^30^,^31^ and theoretically^32^,^33^, with the result that oblate ellipsoids are subject to stronger adhesion than spheres with the same volume. To better understand the adhesion potential of micro- and nano-particles, a quantitative description of particle margination under realistic blood flow conditions is required.

In this work, we investigate the role of particle size and shape on the margination efficiency, and therefore on their adhesion potential. Several sizes ranging from about hundred nanometers to a few micrometers and two different shapes (spherical and ellipsoidal) are considered. The margination of micro- and nano-particles is studied numerically for a wide range of hematocrit values, vessel sizes, and flow rates using a combination of two-dimensional (2D) and three-dimensional (3D) models. Our results indicate that large particles possess a larger probability of being marginated than small particles. As the particle size becomes very small (less than about 100–200 nm), the particle distribution within vessel cross-section can be described well by the plasma volume around flowing RBCs. Furthermore, spherical particles marginate better than ellipsoids, however the adhesion efficiency of ellipsoidal particles is expected to be superior in comparison to that of spheres due to their slower rotational dynamics near a wall. Finally, we discuss what size and shape of micro- and nano-carriers may be best suited for biomedical applications.

## Results

**Figure 1:**
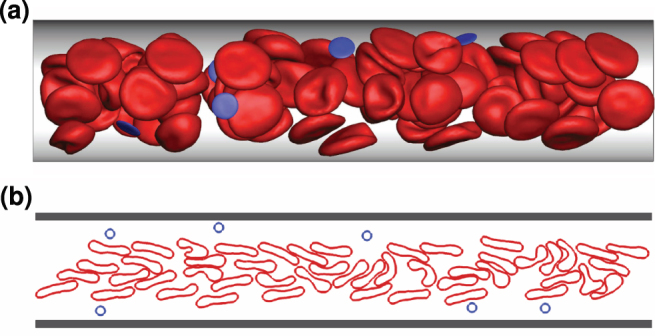
Snapshots of cell and particle conformations in microchannels in 3D and 2D. RBCs are colored in red and suspended particles in blue. (a) 3D simulation snapshot of blood flow for *H_t_* = 0.3 and 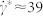. (b) 2D simulation snapshot of blood flow for *H_t_* = 0.3 and 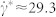.

Blood is modeled as a suspension of RBCs and micro- or nano-particles, while blood flow is studied in idealized microvessels using simulations in 2D and 3D, see Fig. 1 and Methods section for details. The 3D blood model has been shown to properly capture essential properties of blood flow in microchannels^34^ as well as blood rheological characteristics^35^,^36^. To study micro- and nano-particle margination for a wide range of conditions, we also exploit a 2D blood flow model due to its numerical efficiency; however, we will show that the 2D model is able to qualitatively reproduce the required blood flow characteristics and the particle margination effect in comparison with the 3D model.

### Blood flow characteristics

The simulated system corresponds to a cylindrical microvessel in 3D with the diameter *W* or to a channel in 2D with the width *W*. We focus here on channel width *W* = 20 μm, but the cases of *W* = 10 μm and *W* = 40 μm are also discussed. In flow direction, periodic boundary conditions are assumed and blood flow is driven by a constant force applied to all solvent particles, which is equivalent to a prescribed pressure drop. The hematocrit *H_t_* is defined as the volume fraction of RBCs. To characterize the flow strength, we define a non-dimensional shear rate in both 2D and 3D as 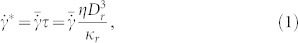
where 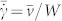 is the average shear rate (or pseudo shear rate) and 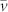 is the average flow velocity computed from the flow rate, while *τ* defines a characteristic RBC relaxation time. Here, *η* is the solvent's dynamic viscosity, 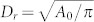 in 3D and *D_r_* = *L*_0_/*π* in 2D are the corresponding RBC diameters, where *A*_0_ is the RBC surface area in 3D and *L*_0_ is the cell contour length in 2D, and *κ_r_* is the RBC membrane bending rigidity. The RBCs are further characterized in 2D by the reduced area 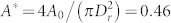, and in 3D by the reduced volume 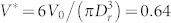, where *A*_0_ is enclosed RBC area in 2D and *V*_0_ is the enclosed RBC volume in 3D. Typical values for healthy RBCs are *D_r_* = 6.5 μm in 3D, while *D_r_* = 6.1 μm in 2D, *η* = 1.2 × 10^−3^ Pa s, and *κ_r_* lies within the range of 50–70*k_B_T* for the physiological temperature *T* = 37°C. Suspended micro- and nano-particles are characterized by the diameter for spheres and by the long axis for disks, denoted *D_p_* in both cases.

### Particle margination in 2D and 3D

**Figure 2:**
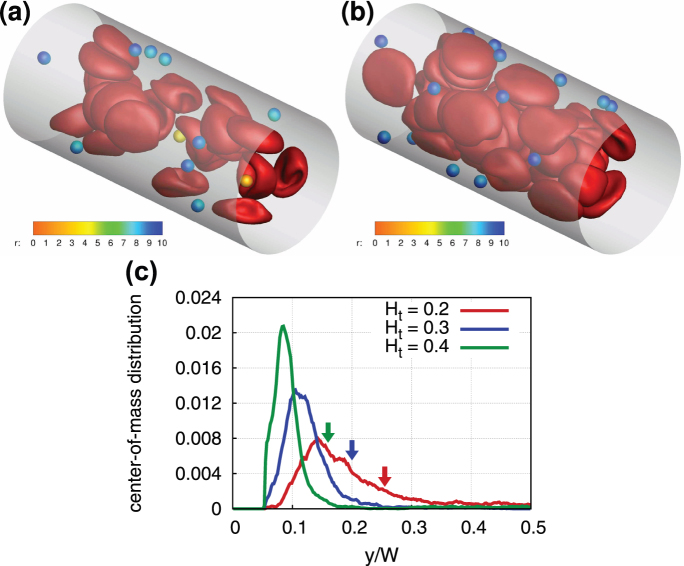
Particle distributions in blood flow. Illustrations of 3D simulations of blood flow for the shear rate of 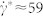 and different hematocrit values (a) *H_t_* = 0.2 and (b) *H_t_* = 0.4. RBCs are drawn in red, while spherical carriers with a size of *D_p_* = 0.28*D_r_* (1.83 μm) are colored according to their radial position *r*. For better contrast, carrier positions from several time instances are superimposed in the plots. (c) Center-of-mass distributions of carriers for various *H_t_* values at 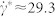. 2D simulation results for circular particles with *D_p_* = 0.3*D_r_* (1.83 μm). The wall is at *y*/*W* = 0. The arrows indicate the boundary of the RBCFL for the different hematocrits, marked by corresponding colors.

Margination of micro- and nano-particles in blood flow depends on *H_t_*, *W*, and 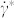. Figures 2(a),(b) illustrate the distribution of carriers of size *D_p_* = 0.28*D_r_* (1.83 μm) for two *H_t_* values in 3D. For better visibility, the carrier positions from a few snapshots are superimposed in the plot. The carrier surfaces are colored according to their radial position in the channel, with yellow color indicating a position near the channel center, while blue color corresponds to a position near the wall. Clearly, the carriers are marginating better for the case of larger *H_t_*.

Carrier positions in blood flow sampled over time lead to particle distributions, which reflect the probability of a particle to be at a certain distance from the wall. Figure 2(c) shows several center-of-mass distributions of circular particles in 2D with *D_p_* = 0.3*D_r_* (1.83 μm) for several *H_t_* values and 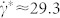. The RBC-free layer (RBCFL) thickness, which is computed from simulation snapshots through the analysis of the RBC core boundary^34^ similar to experimental measurements^37^ (see Supplementary Fig. S1), is depicted by small arrows. The distributions have been averaged over the halves of the channel due to symmetry. Figure 2 shows that the carriers migrate into the RBCFL and remain quasi-trapped there. With increasing *H_t_*, the carriers marginate better, as indicated by the development of a strong peak in the distribution near the wall at *y*/*W* = 0, and the motion of the peak position towards the wall. This is due to a decrease in the RBCFL thickness leading to a smaller available space for the particles. This trend is in agreement with experimental observations^17^ and simulations^38^,^39^,^40^ of margination of blood platelets, which have a comparable size.

**Figure 3:**
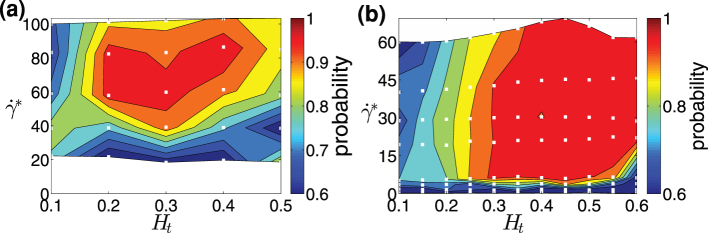
Particle margination in 3D and 2D. Probability diagrams of particle margination with respect to 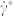 and *H_t_* in (a) 3D and (b) 2D, where the margination probability is defined as a probability of a particle center-of-mass to be within the RBCFL. The white squares (□) indicate the values of *H_t_* and 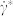 for which simulations have been performed.

To quantify and compare particle margination for a wide range of flow and particle parameters, we define the margination probability as a fraction of particles whose center-of-mass is located within the near-wall layer of thickness *δ*. The choice of *δ* depends on the exact problem to be addressed, and several possibilities can be considered. To describe particle margination into the vicinity of a vessel wall, it is natural to select *δ* to be the RBCFL thickness. Typical values of RBCFL thickness and their dependence on *H_t_* are displayed in Supplementary Fig. S2. Figures 3(a),(b) present margination probability diagrams of particles for a wide range of *H_t_* and 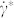 values corresponding to 3D and 2D simulations, respectively; the comparison shows that roughly 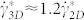. Particle margination strongly depends on *H_t_* as well as on shear rate. At low *H_t_* values, particle margination is expected to be weak, while at high *H_t_* the margination might be also attenuated due to particle-RBC interactions near a wall. The latter effect has been described for a marginating white blood cell^41^ and is expected to subside for particles substantially smaller than a RBC, i.e. of sub-micrometer size. A pronounced dependence of particle margination on shear rate is observed at low flow rates. In the limit of very small flow rates (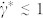), the RBC distribution should be nearly uniform, and therefore, the RBCFL and consequently particle margination should almost vanish. As the shear rate is increased, the RBCFL thickness grows rapidly^42^, leading to a substantial increase in particle margination.

The simulated values of 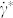 cover the range of flow rates characteristic for the venular part of microcirculation (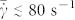 for *W* ≈ 20 μm), where it is estimated that 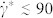 in 3D (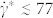 in 2D), while in arteriolar part the flow rates are higher (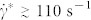 for *W* ≈ 20 μm) with 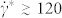 in 3D^43^,^44^. The considered range of shear rates is also relevant for tumor microvasculature, since blood flow velocities in tumors are much reduced in comparison to those under normal conditions, due to high geometric resistance and vessel permeability^45^,^46^. Furthermore, the margination probability diagrams in Figs. 3(a),(b) show that the strongest particle margination occurs in the range of *H_t_* = 0.25–0.6. This region has a considerable overlap with the characteristic hematocrits in the body's microvascular networks in the range *H_t_* = 0.2–0.4. A strong particle margination at high *H_t_* values seems to be an advantage for drug delivery to tumors, since blood within tumor microvasculature is often subject to hemoconcentration due to plasma leakage^47^. We also note that particle margination obtained from 3D simulations displays a higher margination probability at lower *H_t_* values than that in the corresponding 2D system. This difference arises from the variation in RBCFL thicknesses in 2D and 3D systems. Thus, RBCFLs in 3D tubes are thinner than those in 2D channels for the same *H_t_* values due to cylindrical curvature of the geometry, which affects close-packing of flowing RBCs. In order to relate simulations with similar RBCFL thicknesses, 3D margination data should be compared with 2D data at a larger hematocrit (by about 0.1–0.2), see Supplementary Fig. S2. In addition, the 3D data also shows a decrease of particle margination at high shear rates. Nevertheless, both 2D and 3D simulations show qualitatively similar trends for the dependence of carrier margination on *H_t_* and 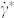. Therefore, we conclude that 2D simulations are able to properly capture particle margination properties in blood flow. Further, we will often employ a 2D system due to its robustness and low computational cost in comparison with a 3D system.

### Dependence of margination on particle size

**Figure 4:**
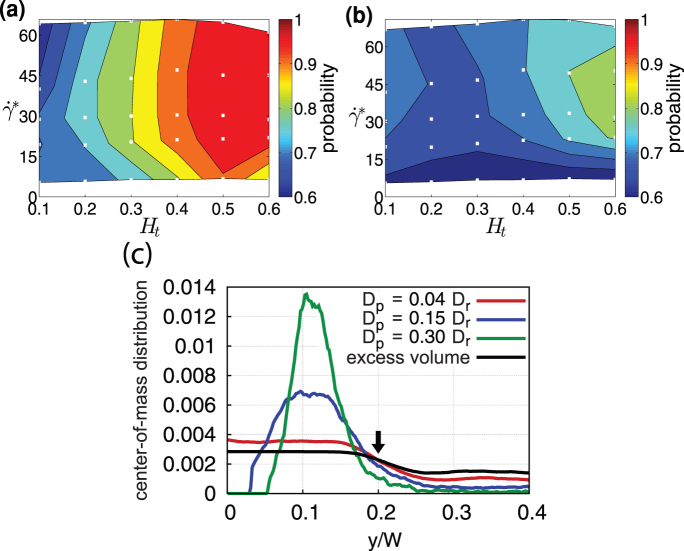
Dependence of margination on particle size. Probability diagrams of particle margination in 2D for various *H_t_* and 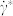 values and for circular particles with the sizes (a) *D_p_* = 0.15*D_r_* (0.91 μm), (b) *D_p_* = 0.04*D_r_* (0.25 μm). The white squares (□) indicate the values of *H_t_* and 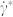 for which simulation were performed. The margination probability is calculated based on the RBCFL thickness. (c) Distribution of particles with different sizes across the channel for *H_t_* = 0.3 and 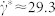. For small particles the distribution resembles the black solid curve computed as the blood-plasma volume. The arrow denotes position of the RBCFL boundary.

The discussion above considered the margination of micron-size particles. There is also a strong interest in nano-carriers, with sizes starting from several nanometers. Figures 4(a),(b) show margination diagrams of particles with *D_p_* = 0.15*D_r_* (0.91 μm) and *D_p_* = 0.04*D_r_* (250 nm), respectively. The comparison of Figs. 4(a),(b) and Fig. 3(b) for *D_p_* = 0.3*D_r_* (1.83 μm) reveals that the region of high margination probability becomes smaller with decreasing particle size. To illustrate the reason for the reduction in margination probability with decreasing particle size, we present in Fig. 4(c) the distributions of particles with different sizes for *H_t_* = 0.3 and 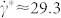. For large enough particles, we observe a pronounced peak in the distribution next to the wall due to their interactions with RBCs, since their size is comparable with the RBCFL thickness. Even though small particles are also marginated, their distribution within the RBCFL is more uniform and their presence around the vessel center line is more probable than that for larger particles. Thus, the cumulative probability for a single particle to be within the RBCFL is lower for nano-carriers than that for micro-particles. Recent *in vivo* experiments^48^ also support our numerical observations that particles with a size of about 1 μm are located closer to the vessel wall than smaller nano-particles. Noteworthy is that the distribution of the smallest particles with *D_p_* = 0.04*D_r_* closely approaches the distribution computed as the excess fluid volume of flowing RBCs. This indicates that the distribution of particles smaller in size than roughly 250 nm can be well approximated by the distribution of the blood plasma, and therefore, their margination properties can be directly inferred from local *H_t_* distributions.

To decide on a suitable particle size for efficient drug delivery, a number of different considerations have to be taken into account. A direct interpretation of probabilities in Figs. 3(b) and 4(a),(b) suggests that larger particle sizes are more favorable for drug delivery due to their better margination properties. To further support this proposition, we consider another definition for the margination probability based on *δ* = 0.5*D_p_* + *s*, which characterizes the fraction of carriers whose closest surface point is not further away from the wall than a distance *s*. We denote such a layer as “potential adhesion layer”, since particle margination into a thin near-wall layer is a necessary precondition for adhesion. Even though the distance *s* is motivated by direct receptor-ligand interactions which occur within several nanometers, resolution restrictions in our mesoscale simulation approach do not allow the selection of smaller distances than approximately *s* = 0.031*D_r_* in 3D or *s* = 0.033*D_r_* in 2D, which corresponds to about 200 nm. Nevertheless, the distance of several hundred nanometers becomes relevant for particle-wall interactions in case of a carrier whose surface is decorated by tethered molecules^49^. Another definition for margination probability can also be based on a fixed layer thickness *δ*, thus it does not depend on *H_t_* or on particle size. Margination diagrams for this definition are illustrated in Supplementary Fig. S3.

**Figure 5:**
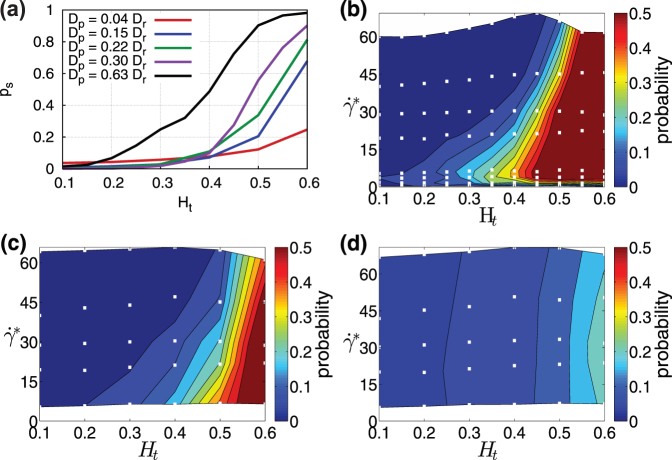
Margination into a potential adhesion layer of thickness 200 nm. (a) Margination probability *p_s_*. The curves correspond to different particle sizes, where *D_p_* = 0.63*D_r_* (3.84 μm) is for an elliptic particle and the other curves are for circular particles. 2D simulation results for 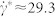. (b–d) Margination diagrams for (b) *D_p_* = 0.3*D_r_* (1.83 μm) (c) *D_p_* = 0.15*D_r_* (0.91 μm), and (d) *D_p_* = 0.04*D_r_* (0.25 μm).

Figure 5(a) presents the margination probability into the potential adhesion layer (*p_s_*) in 2D at 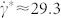. At very small *H_t_*, the fraction of particles within the potential adhesion layer is small for all particle sizes; however, the smallest studied particles seem to be slightly more advantageous here. Remember that the interpretation of 2D margination data for a RBCFL thickness with respect to the same RBCFL thickness in 3D requires a shift in *H_t_* values such that the range of *H_t_* = 0.15–0.4 in 3D corresponds to approximately the range of *H_t_* = 0.3–0.6 in 2D. For the range of *H_t_* = 0.3–0.6, Fig. 5 clearly shows that the fraction of large particles within the potential adhesion layer is much higher than that for small particles. The corresponding margination diagrams are shown in Figs. 5(b–d) and support the conclusion that large particles marginate better for all considered shear rates. This indicates that micro-carriers are likely to be better for drug delivery than sub-micron particles.

### Dependence of margination on vessel size

To elucidate the effect of vessel diameter, we performed a number of simulations in 2D for two additional channel widths (*W* = 10 μm and 40 μm) and two particle sizes (*D_p_* = 0.15*D_r_* and *D_p_* = 0.3*D_r_*); corresponding simulation snapshots are displayed in the Supplementary Fig. S4. We consider margination into both the RBCFL and the potential adhesion layer.

**Figure 6:**
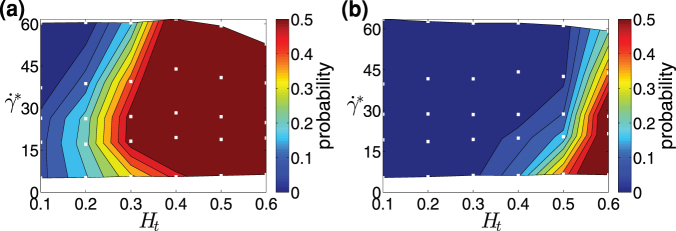
Margination for different channel widths. Margination into the potential adhesion layer based on *δ* = 0.5*D_p_* + 200 nm, for particles with size *D_p_* = 0.3*D_r_* (1.83 μm) and two channel widths (a) *W* = 10 μm and (b) *W* = 40 μm.

The pronounced dependence of particle margination properties on channel width for the potential adhesion layer is illustrated by a comparison of Fig. 5(b) and Fig. 6. For particles with a size of *D_p_* = 0.3*D_r_* (1.83 μm), particle margination into the potential adhesion layer improves considerably as the channel size decreases due to the much smaller RBCFL thickness in narrow channels. Thus, particle adhesion is expected to be more efficient in small vessels (i.e., capillaries) than in large vessels (i.e., venules and arterioles). Supplementary Fig. S5 supports this observation for particles with *D_p_* = 0.15*D_r_* (0.91 μm). Furthermore, a reduction of margination into the potential adhesion layer with decreasing particle size is found for all channel sizes.

Particle margination based on the RBCFL thickness exhibits similar dependence on *H_t_* and flow rate for different channel widths, see Supplementary Fig. S6. For the channel width *W* = 10 μm, margination into the RBCFL differs only slightly for different particle sizes up to *H_t_* ≈ 0.5. For 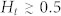, the particle radius might be larger than the RBCFL thickness, leading to an apparent decrease in margination for the large particles. For the cases *W* = 20 and 40 μm, where the RBCFL thickness is always larger than the particle radius, we observe that large particles marginate clearly better than small particles.

### Dependence of margination on particle shape

**Figure 7:**
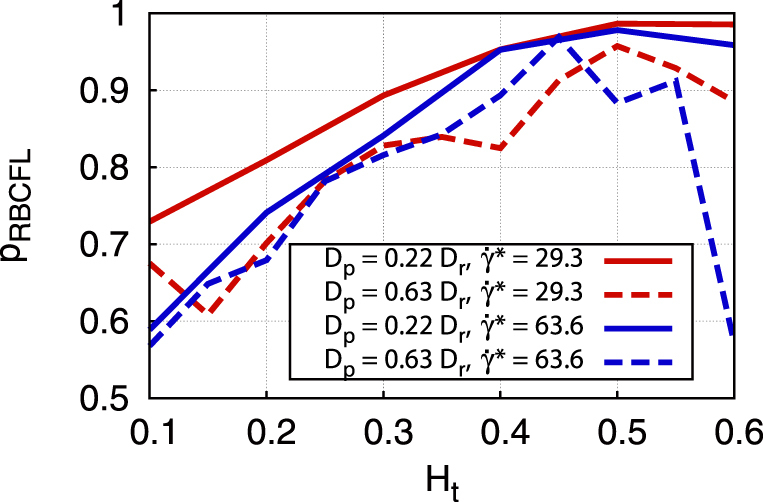
Dependence of margination on particle shape. Margination probabilities of ellipse-like particles (dashed lines) for various *H_t_* and 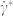 values in comparison to circular particles (solid lines) of the same area. The long axis of a 2D elliptic particle is *D_p_* = 0.63*D_r_* (3.84 μm) and the aspect ratio equals approximately 7. The margination probability is calculated based on the RBCFL thickness.

Advances in micro- and nano-particle fabrication facilitate the production of carriers of various shapes, including spherical, prolate and oblate ellipsoidal, and rod-like shapes^5^. However, advantages of different particle shapes for drug delivery are still to be explored. Thus, we investigate the effect of shape on the margination properties in blood flow. Figure 7 displays results of simulations in 2D for the margination probability (based on the RBCFL) of elliptic particles under various blood flow conditions in comparison to circular particles. The ellipse has an aspect ratio of about 7 and the longest diameter is *D_p_* = 0.63*D_r_* (3.84 μm); the enclosed area corresponds to the area of a circle with diameter *D_p_* = 0.22*D_r_* (1.35 μm). The plot indicates that margination of elliptic particles is slightly worse than that of circular particles. From these data we can also conclude that margination of the elliptic particles with a smaller aspect ratio than 7 is similar to that presented in Fig. 7. However, since the largest diameter of the ellipse is larger than that of a circle with the same area, its margination into the potential adhesion layer, which is defined as a probability of a particle to be within a near-wall layer of thickness *δ* = 0.5*D_p_* + 200 nm, appears to be considerably larger for ellipsoids than that for the corresponding sphere (see Fig. 5). We have also performed a number of 3D simulations with oblate ellipsoids having the same aspect ratio, which showed that their margination is qualitatively similar to that in 2D.

Recent theoretical^32^,^33^ and experimental^30^,^31^ studies suggest that ellipsoidal particles possess better adhesion properties than spheres due to a larger contact area for adhesion interactions. It is also interesting to consider drag force on an ellipsoid or sphere in shear flow near a wall. In case of an ellipsoid close and parallel to a wall, the drag force is found to be smaller than that on a sphere with the same volume, which was estimated in separate simulations of a sphere and an ellipsoid in shear flow with fixed position. Thus, adhered ellipsoidal particles experience a lower drag force due to fluid flow than the corresponding spheres with the same volume. In conclusion, the current knowledge about adhesion of ellipsoidal particles and our simulation results on margination suggest that ellipsoidal particles are very likely a better choice for drug delivery than spherical particles.

### Dynamics of marginated particles

**Figure 8:**
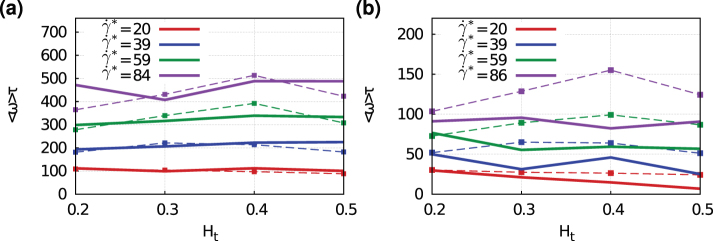
Dynamics of marginated spherical and elliptical particles within the RBCFL. Comparison of average angular velocities 〈*ω*〉 of (a) spherical and (b) ellipsoidal particles for various wall shear rates 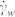 in 3D. The simulation results 〈*ω*〉 (solid lines) are also compared with the theoretical prediction (dashed lines) by Jeffery^50^ for a particle in simple shear flow.

Local particle dynamics may also influence the margination and adhesion efficiency. Simulations in 3D show that the dynamics of marginated particles (i.e., within the RBCFL region) is different for spherical and ellipsoidal particles. A spherical particle is subject to a uniform rotation, while an ellipsoid displays tumbling dynamics. A quantitative analysis of the average angular velocities 〈*ω*〉 of marginated particles shows that ellipsoidal particles rotate considerably slower within the RBCFL than spherical carriers, see Fig. 8. The comparison is made for a sphere and an ellipsoid of the same volume, while the long semiaxis of the ellipsoid is about twice the radius of the sphere. Within the RBCFL region it is plausible to assume a simple shear flow with the wall shear rate 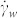, which can be computed directly from a near-wall velocity profile or estimated from the pressure gradient applied to drive the flow. In Fig. 8, we compare the average angular velocities of marginated spherical and ellipsoidal particles with the theoretical predictions by Jeffery^50^ for an oblate ellipsoidal particle in shear flow, which is given by 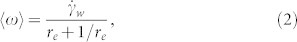
where *r_e_* is the aspect ratio of major and minor axis. For a sphere, *r_e_* = 1 which implies 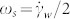, while for an ellipsoid, *r_e_* = 7 which results in 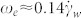, such that *ω_e_* < *ω_s_*. In addition, while the results for 〈*ω*〉 in Fig. 8(a) for a sphere are close to the theoretical results, the computed angular velocities for an ellipsoid in Fig. 8(b) are lower than the corresponding theoretical predictions due to the confinement of the ellipsoid between the wall and flowing RBCs. A lower rotational velocity of a particle leads to a longer interaction time between the particle and a wall. Thus, adhesion of ellipsoidal particles is expected to be more efficient than for spheres with a comparable size. In conclusion, a detailed analysis of dynamics of marginated particles further supports the proposition that ellipsoidal particles are likely to be better candidates for drug delivery.

## Discussion

Particle margination in blood flow depends on particle size and shape, hematocrit, vessel size, and flow rate. Margination of spherical and ellipsoidal particles increases with increasing hematocrit, while their margination properties appear to be rather similar, where a sphere marginates slightly more efficient than an ellipsoid. The presented diagrams show that larger particles have a higher margination probability in comparison to the smaller ones. Moreover, the distribution of very small particles with a diameter smaller than approximately 250 nm is well represented by the blood plasma volume of RBCs. Margination of particles into the potential adhesion layer is found to be more pronounced in small vessels, indicating that particle adhesion is likely to occur more often in capillaries than in arterioles and venules.

The simulation results are in good qualitative agreement with several experimental observations^15^,^17^,^25^,^30^,^31^,^48^. For example, margination of micro-particles has been observed to be more efficient than that of nano-particles in recent *in vivo* experiments^48^. However, a detailed quantitative comparison is still difficult due to two reasons. On the one hand, the majority of the simulation results is obtained for 2D systems, which provide interesting insights into the relevant mechanisms, but have limited power for quantitative predictions for 3D systems. On the other hand, experimental data on particle margination in blood flow^15^,^48^ are very scarce and most of the available experimental investigations (e.g., Refs. 25, 30, 31) focus on carrier adhesion. Even though margination is a necessary pre-condition for particle adhesion to vessel walls, particle margination and adhesion are not equivalent, since carrier adhesion may also depend on other factors (e.g., specific targets, the receptor/ligand density and distribution).

Clearly, the size and shape of drug carriers are important parameters not only for margination, but also for their adhesion and further transport through biological barriers (e.g., internalization). Our simulations suggest that ellipsoidal particles are expected to adhere more efficiently than spherical carriers due to a larger surface for adhesive interactions and decelerated tumbling motion within the RBCFL. Therefore, our future numerical investigations will be focused on the adhesion ability of various particles in blood flow. Further requirements for efficient drug delivery include particle transport through vessel walls, interstitial space, and cell membranes. For instance, particle internalization by endothelial cells and intracellular trafficking have been shown to be most efficient for spherical sub-micron particles, rather than for micron-size carriers with an ellipsoidal shape^10^. This observation points in the direction of smaller carrier to be most efficient for internalization. As a consequence, the concept of multi-stage drug-delivery carriers^1^,^7^, where a larger micro-particle incorporates a number of small nano-carriers, seems to be very promising. In this way, margination and carrier delivery or adhesion to a specific target within the microvasculature could be achieved using micro-particles, which would then be followed by the release of nano-particles into the tissue. In conclusion, tackling various drug-delivery challenges is a complex issue; its resolution requires an inter-disciplinary effort including *in vitro* and *in vivo* experiments and realistic numerical simulations.

## Methods

### Simulation method

We employ the dissipative particle dynamics (DPD) method^51^,^52^ for 2D simulations and the smoothed DPD (SDPD) method^53^ for 3D simulations, where both methods are mesoscopic particle-based simulation approaches which properly capture hydrodynamics. Both simulated systems are represented by a collection of *n* point particles. The particles interact locally within a selected cutoff region through three pairwise forces denoted as conservative (**F***^C^*), dissipative (**F***^D^*), and random (**F***^R^*) forces. The time evolution of the velocity **v***_i_* and position **r***_i_* of particle *i* with the mass *m_i_* is determined by the Newton's second law of motion *d***r***_i_* = **v***_i_dt* and 
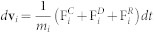
. More information on the DPD formulation can be found in Refs. 51, 52, while the DPD parameters used in 2D simulations are presented in Supplementary Tab. 1.

In the SDPD method, the forces are derived by a discretization of the Navier-Stokes equation similar to the smoothed particle hydrodynamics (SPH) method^54^, while the implementation of thermal fluctuations is analogous to that in DPD^53^. The forces on particle *i* are given by the conservative force 
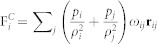
, the dissipative force 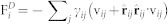, and the random force 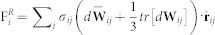. Thereby, *d***W***_ij_* is a matrix of independent Wiener increments and 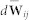 is its traceless symmetric part. Here, *p_i_* and *p_j_* are particle pressures which are given by the equation of state *p* = *p*_0_(*ρ*/*ρ*_0_)*^α^* − *b* with *p*_0_, *ρ*_0_, *α*, and *b* being model parameters, see Supplementary Tab. S2. The particle density *ρ_i_* is calculated locally as 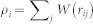, where 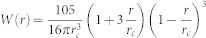 is the Lucy function^54^ and *r_c_* is the cutoff radius. The weight function *w*(*r*) is determined by ∇*W*(*r*) = −**r***w*(*r*). The coefficients *γ_ij_* and *σ_ij_* define the strength of dissipative and random forces. The fluctuation-dissipation theorem to be satisfied requires 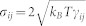. Finally, the friction coefficients are defined as 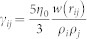.

### Blood components

In 3D, a RBC membrane and suspended carriers are both modeled by a collection of discrete points, which are the vertices of a triangular network of springs on their membrane surface^55^. The network assumes fixed connectivity with the potential energy defined as 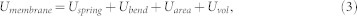
which includes the spring's elastic energy *U_spring_*, the bending energy *U_bend_*, and the area and volume conservation constraints *U_area_* and *U_vol_*. The spring forces mimic the elasticity of a membrane. The bending energy represents the bending resistance of a membrane, while the area and volume energies enforce area-incompressibility of a membrane and incompressibility of the inner cytosol, respectively. Detailed description of these potentials can be found in Ref. 55, while all model parameters are given in Supplementary Tab. S3.

In 2D, RBCs and micro- and nano-particles are modeled as closed bead-spring chains, which incorporate bending rigidity and an area constraint^41^. The model parameters are presented in Supplementary Tab. S4. Carriers in 2D are modeled by a collection of 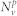 particles (see Supplementary Tab. S5), which are constrained to maintain a rigid configuration.

### Simulation setup

The simulation setup consists of a single channel of cylindrical shape in 3D with diameter *W* = 20 μm and length of *L* = 12.3*D_r_*. In 2D, a slit geometry with different widths *W* = 10, 20, and 40 μm and length *L* = 19.5*D_r_* (independent of *W*) is employed. The channel is filled with fluid particles and with *N* suspended carriers and *N*_RBC_ RBCs. The number of RBCs is computed according to channel hematocrit, which corresponds to the volume fraction of RBCs in 3D and to the area fraction of RBCs in 2D. The number of suspended particles for different simulations is provided in Supplementary Tab. S5.

### Boundary conditions

In the flow direction, periodic boundary conditions (BCs) were imposed, while in the other directions the suspension was confined by walls. The walls are modeled by frozen fluid particles with the same structure as the fluid, while the wall thickness is equal to *r_c_*. Thus, the interactions of fluid particles with wall particles are the same as the interactions between fluid particles, and the interactions of suspended carriers and cells with the wall are identical to those with a suspending fluid. To prevent wall penetration, fluid particles as well as vertices of RBCs and carriers are subject to reflection at the fluid-solid interface. We employed bounce-back reflections, because they provide a better approximation for the no-slip boundary conditions in comparison to specular reflection of particles. To ensure that no-slip boundary conditions are strictly satisfied, we also add a tangential adaptive shear force^56^ which acts on the fluid particles in a near-wall layer of a thickness *h_c_* = *r_c_*.

### Coupling between solvent and cells/carriers

Coupling between the fluid flow and cells/carriers is achieved through viscous friction^55^ between cell vertices and the surrounding fluid particles, which is implemented via the DPD interactions *F^D^* and *F^R^* for both 2D and 3D simulations. The strength *γ* of the dissipative force *F^D^* for the interaction between a fluid particle and a membrane vertex is computed such that no-slip BCs are ensured. The derivation of *γ* is based on the idealized case of linear shear flow over a flat part of a membrane with area *A*. In a continuum hydrodynamics description, the total shear force exerted by the fluid on the area *A* is equal to 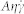, where *η* is the fluid's viscosity and 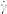 is the local wall shear-rate. The same fluid force has to be also transmitted onto a discrete membrane having *N_A_* vertices within the area *A*. The force on a single membrane vertex exerted by the sheared fluid can be found as 
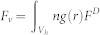
 where *n* is the fluid number density, *g*(*r*) is the radial distribution function of fluid particles with respect to the membrane particles, and *V_h_* is the half sphere volume of fluid above the membrane. Here, the total shear force on the area *A* is equal to *N_A_F_v_*. The equality of 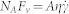 results in an expression of the dissipative force coefficient in terms of the fluid density and viscosity, wall density *N_A_*/*A*, and *r_c_*. Under the assumption of linear shear flow the shear rate 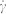 cancels out. This formulation results in satisfaction of the no-slip BCs for the linear shear flow over a flat membrane; however, it also serves as an excellent approximation for no-slip at the membrane surface. Note that conservative interactions between fluid and membrane particles are turned off, which implies that the radial distribution function is structureless, *g*(*r*) = 1. In 2D, the surface area is replaced by a line of length *L* with *N_L_* particles, and the half sphere volume is replaced by the half circle area *A_v_*.

### Gathering statistics

The center-of-mass distributions of particles were calculated on the fly and were written to disk as sub-averages over short time intervals. Final averaging of the data is done during post-processing. To make sure that the final averaging of data starts from a time point which is sufficiently late for the system to be independent of the initial conditions, we have tested the sensitivity of final distributions to the choice of the starting time for averaging.

### Measuring RBC-free-layer thickness

To determine the RBC-free-layer (RBCFL) thickness, we measure the outer edge of the RBC core shown in Supplementary Fig. S1, which is similar to RBCFL measurements in experiments^57^,^37^. The data are averaged for many RBC snapshots at different times. In 3D, the RBC core edge is measured by projecting RBC vertices onto the x-y plane, where curves of the RBC core minimum and maximum are fitted similar to that in Supplementary Fig. S1. Here, we also perform averaging over different angular orientations (to exploit the cylindrical symmetry of the channel) in addition to the temporal averaging.

## Author Contributions

K.M. performed all the simulations and analyzed the computational results; D.A.F. and G.G. designed the research project; K.M., D.A.F. and G.G. interpreted the results and wrote the manuscript.

## Supplementary Material

Supplementary Information Supporting information

Supplementary Information Movie S1

Supplementary Information Movie S2

Supplementary Information Movie S3

Supplementary Information Movie S4
