# Supplementary material for: Margination of micro- and nano-particles in blood flow and its effect on drug delivery
Source: Sci Rep. 2014 May 2;4:4871. doi: 10.1038/srep04871 (PMC4007071; doi:10.1038/srep04871)
Supplement: Supplementary Information — Supporting information [file srep04871-s1.pdf]

SUPPLEMENTARY INFORMATION

Margination of micro- and nano-particles in blood flow and its effect on drug delivery

K. Müller, D. A. Fedosov, and G. Gompper

	m	a	γ	r_c	s	n	k_BT	η
$\dot{\gamma}^* \leq 20$	1	40	10	1.5	0.3	5	1	72.2
$\dot{\gamma}^* > 20$	1	40	20	1.5	0.3	5	1	144.4

Supplementary Table S1. DPD fluid parameters used in 2D simulations. m is the mass of a fluid particle, a and γ are the conservative and dissipative force coefficients, respectively. r_c is the interaction cutoff radius, s is an exponent for the random-force weight function, n is the number density of fluid particles, k_BT is the energy unit with k_B being the Boltzmann constant and T temperature, and η is the fluid's dynamic viscosity. The used notations are the same as in Supplementary Ref. [1].

p_0	ρ_0	α	b	r_c	η_0	n	k_BT	η
100	3.0	7	80	1.5	100.0	3	0.4	107.6

Supplementary Table S2. SDPD fluid parameters used in 3D simulations. The SDPD notations we use are similar to those in Supplementary Ref. [2]. The equation of state is same as in Supplementary Ref. [3]. p_0, ρ_0, α and b are model parameters. η_0 is the desired dynamic viscosity, n is the number density of the fluid, k_BT is the energy unit, and η is the measured (real) fluid viscosity.

	N_v	l_m/l_0	Y/(µN/m)	θ_0	κ/k_BT	A_0/µm^2	V_0/µm^3	$k_d/(k_BT/D_r^2)$	k_a/k_d	k_vD_r/k_d
RBC	500	2.2	18.9	0	70	133	92.5	42250	49	325
Spherical cell	350	2.2	189	0	868	10.5	3.2	422500	50	3250
Ellipsoid	350	2.2	189	θ_0^l	868	24.3	4.3	422500	50	3250

Supplementary Table S3. 3D cell model parameters. All notations here are the same as in Supplementary Ref. [4]. N_v is the number of vertices per cell, l_m is the maximum spring extension, and l_0 is the spring length set to the value of the initially triangulated membrane surface. Y is the Young's modulus, θ_0 is the spontaneous angle between two adjacent faces with θ_0^l being the angles of the initially triangulated shape. The macroscopic bending rigidity κ is given as $\kappa = \sqrt{3}k_b/2$ with k_b being the bending constant. A_0 is the desired cell area and V_0 is the cell volume, while k_d is the local area constraint coefficient, k_a is the global area constraint coefficient, and k_v is the volume constraint coefficient.

	N_v	l_m/l_0	L_0/µm	θ_0	κ/k_BT	A_0/µm^2	$k_a/(k_BT/D_r^2)$
RBC	50	2.2	19.22	0	50	13.6	37210

Supplementary Table S4. 2D RBC model parameters. All notations here are same as in Supplementary Ref. [5]. N_v is the number of vertices forming a cell, l_m is the maximum spring extension, and l_0 is the initial spring length. L_0 is the cell's contour length, θ_0 is the spontaneous angle between two neighboring bonds, κ is the macroscopic bending rigidity and $\kappa = k_bl_0$ with k_b being the bending constant, A_0 is the cell area, and k_a is the area constraint coefficient.

cell size	$D_p = 0.3D_r$	$D_p = 0.63D_r$	$D_p = 0.15D_r$	$D_p = 0.04D_r$
N_v^p	20	20	15	10
$N_{W=20\,\mu\text{m}}$	6	6	20	100
$N_{W=10\,\mu\text{m}}$	6	/	14	/
$N_{W=40\,\mu\text{m}}$	12	/	30	/

Supplementary Table S5. Carrier characteristics. N_v^p is the number of particles per carrier for 2D simulations and N is the number of carriers in the system depending on the channel width and particle size. The numbers of particles in the system are identical for 2D and 3D simulations.

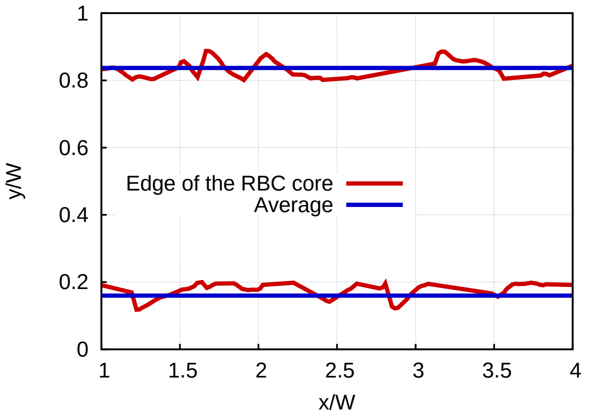

Supplementary Figure S1. A schematic of RBCFL measurement. An example of a RBCFL edge (red line) from a 2D simulation. The blue line presents the average of the RBCFL edge, which defines the RBCFL thickness.

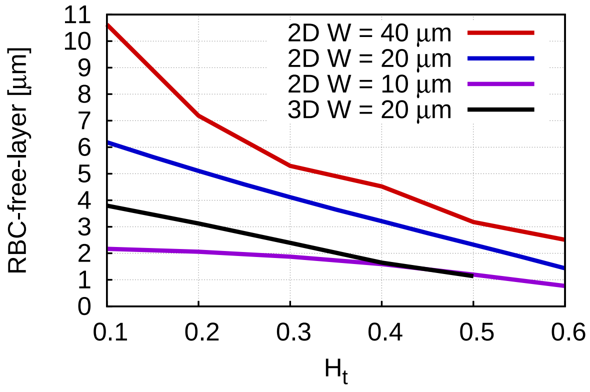

Supplementary Figure S2. RBCFL thickness for various H_t values and channel widths. 2D simulations for $W = 10\,\mu\mathrm{m}$ (purple curve), $W = 20\,\mu\mathrm{m}$ (blue curve), and $W = 40\,\mu\mathrm{m}$ (red curve) at $\dot{\gamma}^* \approx 29.3$. The plot also shows the RBCFL thickness (black curve) in 3D for $W = 20\,\mu\mathrm{m}$ and $\dot{\gamma}^* \approx 40$.

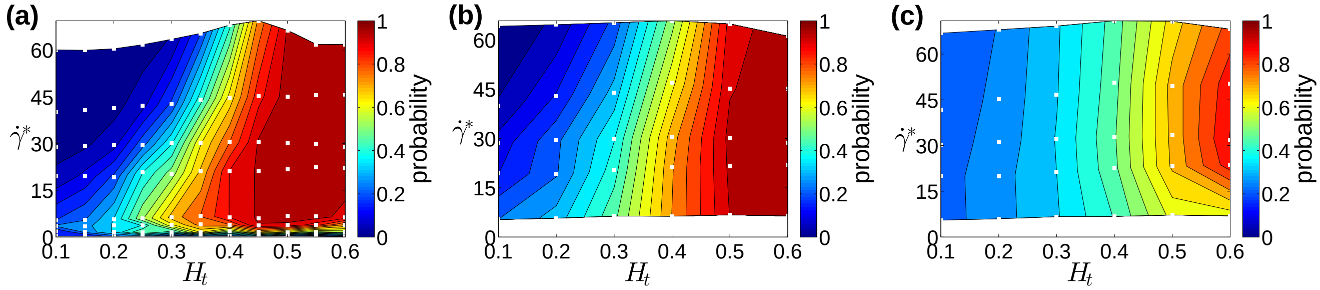

Supplementary Figure S3. Margination of particles into the layer of a fixed thickness. Margination diagrams for various particle sizes: (a) $D_p = 0.3D_r$ (1.83 µm), (b) $D_p = 0.15D_r$ (0.91 µm), and (c) $D_p = 0.04D_r$ (0.25 µm). The margination probability is based on the fixed $\delta = 2\,\mu\mathrm{m}$. 2D simulations for $W = 20\,\mu\mathrm{m}$. The white squares (□) indicate the values of H_t and $\dot{\gamma}^*$ for which simulations were performed.

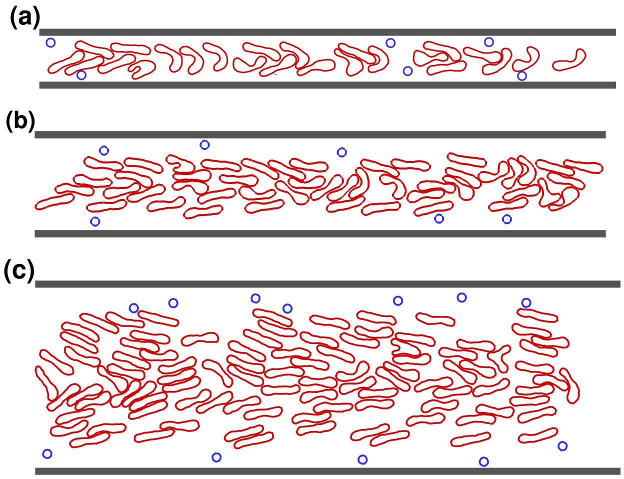

Supplementary Figure S4. 2D simulation snapshots of blood flow for different channel widths. The illustrated snapshots are made for $H_t = 0.3$ and $\dot{\gamma}^* \approx 29.3$. (a) $W = 10\,\mu m$, (b) $W = 20\,\mu m$, and (c) $W = 40\,\mu m$.

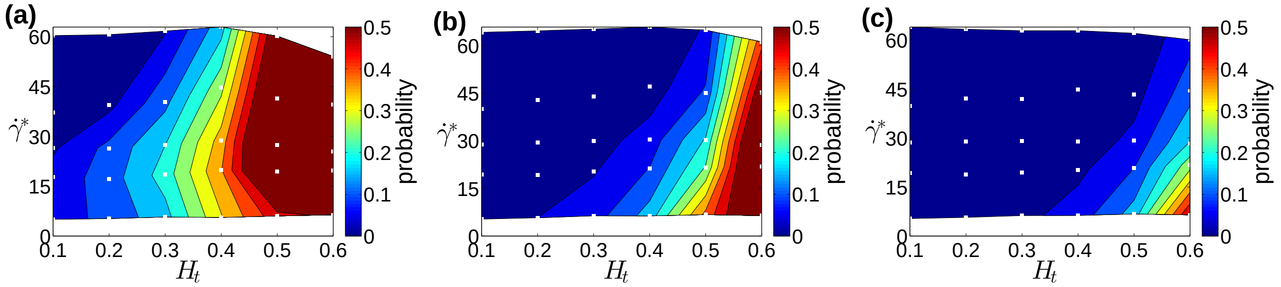

Supplementary Figure S5. Margination into the potential adhesion layer for different channel widths. Margination diagrams are based on $\delta = 0.5D_p + 200\,\text{nm}$ for the carriers of size $D_p = 0.15D_r$ (0.91 µm) and three channel widths. The white squares (□) indicate the values of H_t and $\dot{\gamma}^*$ for which simulations were performed. (a) $W = 10\,\mu m$, (b) $W = 20\,\mu m$, and (c) $W = 40\,\mu m$.

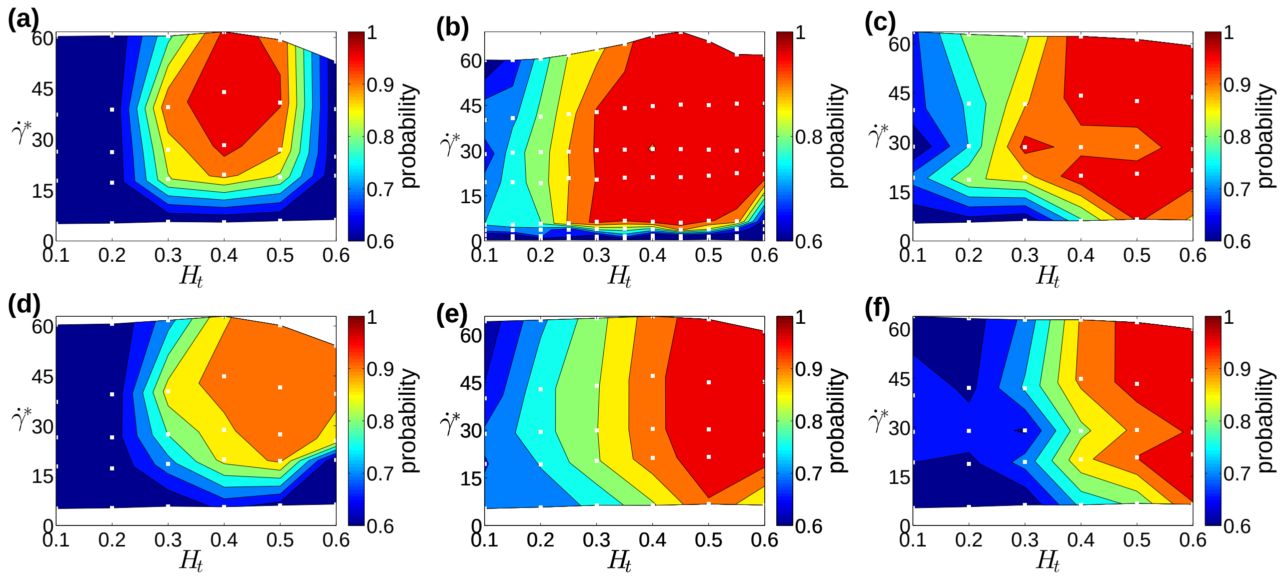

Supplementary Figure S6. Margination into the RBCFL for different channel widths. The diagrams are based on δ = RBCFL thickness for two sizes of carriers and three channel widths. The white squares (□) indicate the values of H_t and $\dot{\gamma}^*$ for which simulations were performed. (a) $D_p = 0.3D_r$ (1.83 µm), $W = 10$ µm, (b) $D_p = 0.3D_r$, $W = 20$ µm, (c) $D_p = 0.3D_r$, $W = 40$ µm, (d) $D_p = 0.15D_r$ (0.91 µm), $W = 10$ µm, (e) $D_p = 0.15D_r$, $W = 20$ µm, and (f) $D_p = 0.15D_r$, $W = 40$ µm.

Supplementary movies

The movies illustrate the dynamics of RBCs and micro- and nano-carriers in flow for 2D and 3D simulations. Only a small segment of the whole simulated channel is displayed.

Movie S1 (circle.mov): The movie shows the 2D flow of RBCs and circular carriers for $H_t = 0.3$ and $\dot{\gamma}^* = 29.3$.

Movie S2 (ellipse.mov): The movie shows the 2D flow of RBCs and elliptical carrier for $H_t = 0.3$ and $\dot{\gamma}^* = 29.3$.

Movie S3 (sphere.mov): The movie shows the 3D flow of RBCs and spherical carriers for $H_t = 0.3$ and $\dot{\gamma}^* = 39$.

Movie S4 (discoid.mov): The movie shows the 3D flow of RBCs and oblate carrier for $H_t = 0.3$ and $\dot{\gamma}^* = 39$.

Supplementary References

[1] Fan, X., Phan-Thien, N., Chen, S., Wu, X. & Ng, T. Y. Simulating flow of DNA suspension using dissipative particle dynamics. *Phys. Fluids* **18**, 063102 (2006).

[2] Vázquez-Quesada, A., Ellero, M. & Español, P. Consistent scaling of thermal fluctuations in smoothed dissipative particle dynamics. *J. Chem. Phys.* **130**, 034901 (2009).

[3] Litvinov, S., Ellero, M., Hu, X. & Adams, N. A. Self-diffusion coefcient in smoothed dissipative particle dynamics. *J. Chem. Phys.* **130**, 021101 (2009).

[4] Fedosov, D. A., Caswell, B. & Karniadakis, G. E. A multiscale red blood cell model with accurate mechanics, rheology, and dynamics. *Biophys. J.* **98**, 2215–2225 (2010).

[5] Fedosov, D. A., Fornleitner, J. & Gompper, G. Margination of white blood cells in microcapillary flow. *Phys. Rev. Lett.* **108**, 028104 (2012).
